# A UK-wide survey of community forensic services for adults with intellectual disability and/or autism

**DOI:** 10.1192/bjo.2024.734

**Published:** 2024-08-20

**Authors:** Iain McKinnon, Ellen Whitehouse, Melissa Harris, Vlad Ciausu, Jane McCarthy, Rory Sheehan

**Affiliations:** Secure Services, Cumbria Northumberland Tyne and Wear NHS Foundation Trust, Newcastle upon Tyne, UK; and Population Health Sciences Institute, Newcastle University, UK; School of Psychology, Newcastle University, UK; Secure Services, Cumbria Northumberland Tyne and Wear NHS Foundation Trust, Newcastle upon Tyne, UK; Learning Disability Service, Sussex Partnership NHS Foundation Trust, Worthing, UK; and Institute of Psychiatry, Psychology & Neuroscience, King's College London, UK; Department of Forensic and Neurodevelopmental Sciences, Institute of Psychiatry, Psychology & Neuroscience, King's College London, UK; and Oxleas NHS Foundation Trust, Dartford, UK

**Keywords:** Psychiatry and law, neurodevelopmental disorders, intellectual disability, autism spectrum disorders, criminal justice system

## Abstract

**Background:**

Specialist forensic community teams for people with intellectual disability and/or autism have been developed, but little is known about their extent and delivery.

**Aims:**

To describe specialist forensic community teams for people with intellectual disability and/or autism across the UK.

**Method:**

An online survey was sent to representatives of each UK Trust/Health Board providing adult mental health and/or intellectual disability services. Questions covered the availability, structure and activities of specialist community forensic services. Quantitative data were summarised and associations between access to specialist forensic teams and care were tested with Chi-squared tests. Thematic analysis of free-text survey responses was used to understand the challenges of providing community forensic mental health services for this group.

**Results:**

A total of 49 out of 78 (63%) eligible Trusts/Health Boards responded, of which 25 (51%) had access to a specialist forensic community team. Teams operated either as part of a single Trust/Board (*n* = 13) or over a larger regional footprint (*n* = 12). The availability of specialist forensic community teams was associated with better access to offence-related interventions (χ^2^ = 15.1002, *P* < 0.005) and co-production of patient care plans (χ^2^ = 7.8726, *P* = 0.005). Respondents reported a wide variation in availability, expertise and perceived quality of community services. The availability of secure and generic in-patient beds, commissioning and legal barriers were also significant challenges in providing appropriate care.

**Conclusions:**

Coverage of specialist community forensic teams is not universal. There are indications that such teams are associated with improved care processes, but further work is needed to establish longer-term outcomes and the optimal model of care.

Community forensic mental health services were developed in the 1990s to improve access to specialised mental healthcare for those who had offended or were considered at high risk of harm to others, but the evidence for their effectiveness is limited.^1^ For people with intellectual disability or autism, specialised community forensic services are a more recent development,^2^ partly in response (in England) to Transforming Care,^3^ a national policy that aims to ensure that people with intellectual disability and/or autism are cared for primarily in the community rather than in hospital. Transforming Care is, in effect, the continuation of the de-institutionalisation movement that began in the 1980s.^4^ A proposed national service model, known as Building the Right Support,^5^ was developed to support the Transforming Care programme and included nine key principles, one of which was the establishment of specialist forensic support in the community for those at risk of engaging in offending behaviours, including supporting the safe discharge of those currently in hospital. However, Building the Right Support did not provide guidance as to how such teams should be configured or commissioned. The development of such teams has also been hampered by a limited evidence base, with methodological constraints and studies employing inconsistent definitions of offending behaviour.^6^ Furthermore, policies appear to consider people with intellectual disability and autism spectrum conditions to require the same approaches to assessment and management; although both fall under the umbrella for neurodevelopmental disorders and there is crossover between the populations, there are also important differences that must be taken into account when providing care.^7^

In response, specialist forensic community teams for people with intellectual disabilities and/or autism have been set up through local initiatives across the National Health Service (NHS) in England,^3^ and little is known about the nature of these services across the UK, including how they deliver care, the workforce and their effectiveness. The aim of this national survey is therefore to describe the current structure and delivery of specialist forensic community teams for adults with intellectual disability and/or autism across the UK.

## Method

### Study design and participants

An online questionnaire comprising nine core questions and branches was devised by the project team to gather information on community mental health services provided for adults with intellectual disability and/or autism and forensic needs (see Supplementary Material available at https://doi.org/10.1192/bjo.2024.734 for the full version of the questionnaire). The questionnaire was designed and piloted by senior psychiatric clinicians from three geographically diverse forensic developmental services, alongside psychiatry and psychology trainees and research assistants. The senior clinicians were all members of the Royal College of Psychiatrists Forensic Intellectual Disability subgroup, and have doctoral research qualifications.

Questions were developed from key components of the Health Education England workforce framework, which, although developed for use in England, is equally applicable to other nations of the UK.^8^ The questionnaire covered the availability of specialist forensic services, staff complement, the model of care (e.g. whether a team assumes statutory responsibility for patients or provides consultation services only) and elements of care provided. The questionnaire comprised a mixture of tick-box items, selection lists and open-ended questions. The questionnaire was hosted on the Joint Information Systems Committee online survey platform (https://www.onlinesurveys.ac.uk/). The project was discussed with and supported as a service evaluation by the Royal College of Psychiatrists Forensic Intellectual Disability working group.

An email containing information about the study and a link to the online survey was sent to representatives of 52 Mental Health Trusts in England, 14 regional Health Boards in Scotland, seven Local Health Boards in Wales and five Health and Social Care Trusts in Northern Ireland. These organisations were selected as they provide NHS adult mental health or intellectual disability services. Professional networks and internet searches were used to identify key contacts in each organisation. In the case that email addresses of individuals were not available, we used a generic or team email address and asked that a relevant person complete the survey.

An initial email was sent to recipients in December 2022 from the Royal College of Psychiatrists Forensic Intellectual Disability subgroup email address. We kept records of responses and sent reminders to organisations that had not responded. The survey was open between December 2022 and March 2023.

### Analysis

Data were stored securely within the online survey system. Simple descriptive statistics were used to summarise the survey data as counts and percentages. The chi-squared test was used to test associations between the presence of a specialist forensic community team and care processes (e.g. co-production of care plans), and between the structure of the team (e.g. a regional or local team) and the team responsibilities (e.g. whether the team held care coordination responsibility or managed a crisis). Analysis was completed in Microsoft Excel 365 for Windows and using an online statistics calculator (www.socscistatistics.com). *P* < 0.05 was considered statistically significant.

The final question on the survey asked participants to comment upon any specific challenges pertaining to the delivery of specialist community services for people with intellectual disability and/or autism who offend. Responses were collated and analysed with thematic analysis.^9^ Responses were anonymised by allocating a response indicator comprising of a letter and number (described below).

Thematic analysis involved four steps: step 1, gaining an understanding of the content of the responses; step 2, identifying and labelling relevant segments of data (coding); step 3, development of themes by grouping similar codes together to develop initial themes, using an iterative process; and step 4, making sense of the themes in relation to the research question (interpretation).

All codes were generated, and initial themes were reviewed, through a collaborative and iterative process by I.M., E.W., V.C. and M.H. Where there was disagreement between team members about coding or allocation to themes, these were discussed and consensus reached. Higher-level themes were then developed from the initial themes by I.M., J.M. and R.S., with the reallocation of initial themes and codes where necessary. This collaborative approach was taken to expand the range of concepts developed, and bring multiple experiences to interpretations of the data. Finally, overarching narratives were developed to encapsulate and describe the data.

### Ethics and consent

This project was carried out as a service evaluation to define and understand current service provision. We used the NHS Health Research authority decision support tool to confirm that no specific ethical approval was required (http://hra-decisiontools.org.uk/research/. All participants gave consent via the online survey. No specific services are identified in this project and no identifiable data were collected, aside from the name of the NHS organisation.

All participants consented to take part in this online survey and provided anonymous responses based on their knowledge of the current service offer in their locality. No personal data or patient identifiable information were collected.

## Results

### Survey response rate

Complete responses were received from 49 of the 78 eligible Trusts/Health Boards (63% overall response rate). This included responses from 33 eligible Trusts in England (response rate 64%), 12 Health Boards in Scotland (response rate 86%), three Health Boards in Wales (response rate 38%) and one Trust in Northern Ireland (response rate 20%).

### Availability of specialist forensic services

Twenty-one (43%) of the 49 Trusts/Boards that responded provided specialist secure in-patient services (at any level of security) for people with intellectual disability or autism. Of those Trusts/Boards that did not provide specialist secure in-patient services, 11 provided specialist ‘sub-secure’ beds (that is, in Assessment and Treatment Units or as locked rehabilitation wards) and 17 (35%) had no specialist in-patient provision for adults with intellectual disability or autism.

Twenty-five (51%) Trusts/Boards had access to a specialist intellectual disability or autism community forensic team (Table 1). We observed that those Trusts/Boards that had specialist secure in-patient services were more likely to have a specialist community forensic team, although this association was not statistically significant (χ^2^ = 3.6001, *P* = 0.06).

**Table 1:** Specialist community forensic services for people with intellectual disability and/or autism and model of care, by UK country

Country	Does Trust/Board have access to SCFS?	Trust/Board versus regional team footprint^b^	Local specialist secure in-patient service^b^	Model of care provided^a^			
				Advice/consultation	Direct work with patients	Care coordination	Crisis management responsibility
England	Yes = 19 (57.6%)	Trust (*n* = 7)	12	7	7	5	3
		Regional (*n* = 12)		12	12	5	9
	No = 14 (42.4%)						
Scotland	Yes = 5 (41.7%)	Board (*n* = 5)	2	5	5	5	4
		Regional (*n* = 0)		−	−	−	−
	No = 7 (58.3%)						
Wales	Yes = 0 (0%)	−	−	−	−	−	−
	No = 3 (100%)	−	−	−	−	−	−
Northern Ireland	Yes = 1 (100%)	Trust (*n* = 1)	0	1	1	1	1
		Regional (*n* = 0)		−	−	−	−
	No = 0 (0%)						
Total	Yes = 25 (51.0%)	Local (*n* = 13)	14	13	13	11	8
		Regional (*n* = 12)		12	12	5	9
	No = 24 (49.0%)						

SCFS, specialist community forensic service.

a. Trust in England and Northern Ireland, Board in Scotland and Wales.

b. Those with specialist forensic community teams only (*n* = 25).

### Structure and model of care of specialist community forensic teams

Of the 25 Trusts/Boards that had access to a specialist community forensic team, 13 (52%) were delivered by a single Trust/Board and the remainder (48%) were regional teams that spanned more than one Trust/Board. The workforce composition of the specialist teams is shown in Table 2.

**Table 2:** Workforce of specialist community forensic teams for people with intellectual disability and/or autism

Profession	Number of specialist teams (%)
Community psychiatric nurses	24 (96)
Psychologist	24 (96)
Psychiatrist	23 (92)
Occupational therapist	18 (72)
Speech and language therapist	17 (68)
Social worker	11 (44)
Specialist support worker	11 (44)
Other^a^	4 (16)
Peer support worker	3 (12)
Dietician	0 (0)

a. Other included art therapist, training coordinator and positive behaviour support practitioner.

All of the specialist community forensic teams provided advice and consultation to other professionals and teams, and all also worked directly with patients to complete assessments or deliver interventions. Sixteen (64%) of the specialist teams reported that they had statutory care coordination responsibility for at least some of the patients open to the team. Having a care coordination role was more likely in a team that was based in a single Trust/Board than a team that worked over a larger, regional area (χ^2^ = 4.9958, *P* = 0.025).

### Care delivery

A range of approaches were used to assess risk across organisations, and most approaches were used by most organisations (Table 3).

**Table 3:** Approaches to risk assessment and management used in community forensic intellectual disability/autism teams

Mechanism	Yes/used (%)	No/not used (%)
Multidisciplinary team reviews	47 (95.9)	2 (4.1)
Structured risk assessment	42 (85.7)	7 (14.3)
Care programme approach	43 (87.8)	6 (12.2)
Case conference review meetings	38 (77.6)	11 (22.4)
Multi-agency meetings (e.g. MARAC, MAPPA)	48 (98.0)	1 (2)
Care and treatment reviews^a^	27 (81.8)	6 (18.2)

MARAC, Multi-Agency Risk Assessment Conference; MAPPA, Multi-Agency Public Protection Arrangement.

a. Denominator for Care and Treatment Reviews is teams operating in England only (*n* = 33).

We asked respondents to indicate whether care plans were co-produced with the patient. Overall, care plans were co-produced with patients in 32 (65%) of services that responded and were not co-produced in the remainder. Trusts/Boards that had a specialist forensic community team (whether holding care coordination responsibility or not) were significantly more likely to co-produce patient care plans than Trusts/Boards that did not have a specialist team (χ^2^ = 7.8726, *P* = 0.005). Respondents could expand on the processes for including patients in co-producing their care plans; results indicated a range of structured and semi-structured tools were used (e.g. DIALOG+, My Shared Pathway, Recovery Star, What I Need) alongside informal strategies such as individual meetings with patients before multidisciplinary reviews to ensure their views are heard.

Thirty-nine (71%) Trusts/Boards provided offence-related psychotherapeutic interventions for people with intellectual disability and/or autism and forensic needs in the community. Those Trusts/Boards with specialist forensic teams were significantly more likely to provide offence-related psychotherapeutic interventions than those without specialist teams (χ^2^ = 15.1002, *P* < 0.005). A slightly higher number of Trusts/Boards, 43 (78%), provided psychotherapeutic interventions for mental health conditions. There was no association between whether a Trust/Board had access to a specialist forensic team and the availability of psychotherapeutic interventions for mental health conditions (χ^2^ = 0.1908, *P* < 0.662). Different teams provided the psychotherapeutic interventions, including the specialist forensic community team, the mainstream forensic community team and the intellectual disability community team, with some overlap within Trusts/Boards. In four Trusts/Boards, no psychotherapeutic interventions were available to people with intellectual disability and/or autism and forensic needs; all of these Trusts/Boards lacked access to a specialist forensic team.

There were varied mechanisms for responding to patients in crisis in the Trusts/Boards that had a specialist forensic community team for people with intellectual disability and/or autism. Some (*n* = 6) specialist teams assumed management responsibility in such cases, whereas others (*n* = 5) provided support for other teams (e.g. the intellectual disability or generic forensic community team) to manage the crisis situation short of taking a lead in the case. In a number of cases (*n* = 11), the specialist team provided both management responsibility and support. Three specialist forensic services indicated that ‘other’ arrangements were in place for management of patients in crisis. Those teams that held care coordination responsibility were more likely than specialist teams that did not offer care coordination responsibility to manage a crisis situation (rather than only provide support or no input), but this relationship was not statistically significant (χ^2^ = 3.5858, *P* = 0.058).

### Thematic analysis

**Fig. 1:**
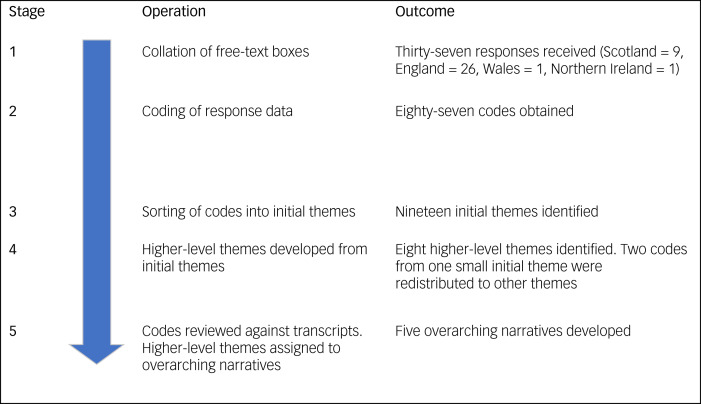
Stages of the thematic analysis.

Thirty-eight organisations left comments in the final free-text section, mainly from England (*n* = 26) and Scotland (*n* = 10). There was only one response each from Wales and Northern Ireland, and each cited a lack of specialist services within their respective geographical locales. The remaining 36 response texts were allocated an anonymised name (i.e. E + number for English NHS Trusts or S + number for Scottish Health Boards). Initial coding of the responses generated 86 codes. These were then arranged into 19 initial themes. Following review using an iterative process eight higher themes emerged, plus one miscellaneous theme. The two codes within this theme were reallocated to other themes. Eight higher-level themes were then reviewed again by I.M., J.M. and R.S., and the five following overarching narratives emerged (see Fig. 1).

#### Narrative 1: variation in the provision of specialist community forensic teams and interagency working

The provision of specialist community forensic services for people with intellectual disability and/or autism varies enormously across England and Scotland. In England, although some NHS Trusts provide their own specialist forensic services, the introduction of supra-regional Provider Collaboratives has led to some ‘lead Trusts’ providing specialist services across a collective of Trusts. Nevertheless, even larger Trusts told us that they are ‘inadequately resourced for the needs of this population’ (E5) with ‘variance across the geographical area in terms of (provision)’ (E3). One respondent reported that ‘discharges will be safer’ (E6) because of specialist input, but others expressed concern that some of the specialist services ‘would discharge (patients) from (their) caseload after 3–6 months’ (E1), often with no suitable recipient team. There was also concern expressed about some of the specialist teams who ‘do not provide care co-ordination/case management’ (E24).

Variability in the functioning of specialist teams was also encountered among Scottish NHS Boards. One respondent reported that ‘integrated working is well established with Care Management, Criminal Justice Social Workers (CJSW), Courts and health professionals’ (S9) across neighbouring health boards. However, another cited a lack of ‘pathways (and) protocols, with the exception of patients that are within MAPPA’, suggesting that only the very riskiest patients with prior convictions were receiving an enhanced level of care and supervision (S2).

Many areas reported no dedicated specialist provision, and this was especially encountered where case numbers were small because of geographical considerations (S1), leading to huge challenges in managing crises (S8) or being able to repatriate patients to their home areas (E14).

#### Narrative 2: availability and willingness of non-forensic community clinical teams to support people with intellectual disability who offend

As specialist community forensic services for people with intellectual disability and/or autism are neither ubiquitous nor all-inclusive, there is often a need for existing intellectual disability community mental health services to provide support for people with intellectual disability who are at risk of offending. There was an evident mismatch in provision of community services for such patients, and possibly a culture of exclusion, with some generic intellectual disability community services ‘declining to work with ‘forensic patients’ citing risk issues’ (E5) or ‘providing … for people with a moderate-severe LD [learning disability] and supporting mainstreaming of other(s)’ (E2). This risks excluding most people with intellectual disability who display offending behaviour who tend to be in the mild-borderline intellectual disability range.

For patients unable to access community intellectual disability services (e.g. those with autism but no intellectual disability), access to nearby community forensic mental health services was of limited use. One respondent found that ‘attempting to push such patients to generic forensic teams (is) problematic, especially in cases where (there is) no mental illness’ (E21). Another problem with generic services was the ‘misunderstanding (the) clinical needs of (the) LD [learning disability] population in particular those with (a) forensic history and absence of … mental … illness’ (S6). As well as a perception that forensic mental health services struggle to understand the support needs of people with intellectual disability, one respondent reported a lack of ‘provision for (specialised offence) treatment work in the mainstream (providers)’ (E10), thus highlighting a paucity of preventive treatment programmes for those who have already offended or are at risk of doing so. It was promising to hear that some generic forensic community services were ‘more aware of autism than before’ but nevertheless ‘appear to have little skill in supporting people (with autism)’ (E6).

#### Narrative 3: provision of quality community placements

Some respondents had encountered substantial problems with the quality and suitability of community placements for their patients. Some thought they were ‘very expensive’ (E22) or lacking ‘suitably adapted living spaces’ (S5). There is also a problem with ‘long lead in time(s) for developments to be available’ (E4), which prevents the timely discharge of people no longer requiring in-patient treatment, but who still require tailored support to effect a safe discharge. One respondent described difficulties developing new services resulting from a lack of existing providers with the requisite skills and experience resulting from ‘long standing commissioning gaps’ (E7). Respondents also told us that once in the community, some patients continue to face barriers such as being ‘excluded from (community) groups due to (their) forensic history’ (S8) and the perceived risks this carried with it, such as having more free time/risk of boredom related to additional offending.

The ‘leadership and consistency of managers (and) the relationships built up’ (E4) were also considered vital factors in providing long lasting and robust placements for patients in the community. Furthermore, it is necessary to be able to access ‘alternative accommodation options in the community to avoid unnecessary hospital admission - particularly at times of crisis’ (E3).

One respondent described that the provision of services ‘based upon evidence-based pathways is lacking locally and needs to be picked up by the (integrated care boards (ICBs))’ (E25). Another reported that a lack of engagement by local authorities and NHS commissioners prevented discharge, especially when there was poor provision in particular localities: ‘There is (sic) a lot of discussions that often lead nowhere because of the poor provision in other areas to support offenders with intellectual disability and an absence of willing by community services, Local Authorities and ICBs to make it happen’ (E6).

#### Narrative 4: availability of in-patient services when an admission is required

Scotland-based respondents cited a lack of ‘access to beds in a crisis’ (S6) and a large reliance on distant ‘Scottish Regional Services (or) private settings in England’ (S7). The reliance on general adult psychiatry beds was highlighted across both nations, with hospital stays ‘cut short with (the) emphasis always placed on discharge’ (S6). This also led to challenges for specialist community forensic services for people with intellectual disability and/or autism being able to ‘identify and discharge (a) person from a … generic in-patient bed’; this is reported to arise from clinicians in generic in-patient services being unfamiliar with the needs of this population or not appreciating the utility of specialist community teams where they exist (E4).

Two England-based respondents cited concerns about risks associated with recent policies leading to bed closures; these were felt to have led to ‘delays in admitting people in risky situations, which feel like a Transforming Care hangover … ’ and that ‘ … people (are) unwilling to have conversations about admissions’ (E22). Another respondent described the situation of ‘significant pressure to raise thresholds for secure hospital admission and to discharge service users when their therapeutic work is not adequately completed’ (E5). The same respondent was also fearful about the consequences of fewer specialist secure in-patient beds: ‘We are now seeing increasing numbers of vulnerable service users with LD [learning disability] and/or autism ending up in prison in environments where reasonable adjustments are inadequate to keep them safe, and they cannot access appropriate therapeutic interventions, due to lack of appropriate hospital beds and the approach that this group “should not” be admitted to hospital’ (E5).

#### Narrative 5: impact of criminal justice interfaces and legal barriers

One respondent was concerned that ‘changes in staffing within Criminal Justice Social Work can provide a challenge due to difficulties in recognition when the individual presenting to court may have intellectual disability or autism spectrum disorder’ (S9), meaning that some of the most vulnerable people may not be identified. Another reported a barrier to effective community support on the release of individuals from prisons ‘challenge of trying to provide input into prisons because the service user may be placed in a prison hours from their home area’ (E5).

One respondent told us that ‘We have some challenges supporting people who are not subject to any legal frameworks … but frequently reoffend and serve prison sentences (stuck in an offending cycle yet may not be “divertible” to in-patient forensic units)’ (E9), highlighting the lack of a legal framework to compel treatment in the community, as well as the lack of in-patient beds meaning that proactive risk management and treatment is not available. Another described that ‘there are more individuals, who are restricted via (the) MM legal ruling’ (E4); this is a legal issue specific to England and Wales, which holds that patients with capacity cannot be conditionally discharged with conditions that amount to a deprivation of liberty. Consequently, there is an increased necessity for specialist forensic intellectual disability teams to support complicated leave and discharge arrangements.

#### Other important factors related to groups with special characteristics

Although a small number of responses, the analysis revealed important themes relating to women and transgender patients. One respondent told us that ‘in (the) region there are no specialist services for women with LD [learning disability] or autism. It is also increasingly clear that service users who are transgender are not being appropriately catered for within secure hospital settings’ (E5) or, for that matter, in the community. Another said, ‘we have autistic women in the in-patient service who pose big challenges in terms of supporting them as in-patients and in discharging them to a supportive, safe care package in the community’ (E6).

## Discussion

### Summary of findings

There continues to be pressure on specialist forensic intellectual disability bed numbers across the UK, either as historical artefact as in Scotland^10^ or because of government policy in England.^11^ As more people with intellectual disability and/or autism move from hospital into the community, so the need has grown to provide safe and effective community forensic services for this population. The commissioning of specialist community forensic services for people with intellectual disability and/or autism is currently an issue specific to national policy in England,^5^ although provision of such specialist services has relevance to all parts of the UK. The aim of this study was to investigate the coverage of specialist community forensic teams for people with intellectual disability and/or autism across the UK, to provide an overview of their structure and functions, and to gather the views and experiences of senior clinicians in these services around the unique challenges of providing care in this context.

The detailed feedback from services highlighted a number of key narratives, including the wide variability of service provision, reluctance of non-forensic teams to provide support to offenders, lack of suitable community placements, the impact of in-patient bed closures and substantial issues where criminal justice and legal systems interface with healthcare delivery.

### Availability and nature of specialist service models

In early 2023, just half of the Trusts/Boards in the UK that responded to our survey had access to a specialist community forensic team for people with intellectual disability and/or autism. Access to specialist teams was more likely in Trusts/Boards that also provided specialist in-patient secure services, suggesting that those organisations with a tradition of specialist forensic care have been more able to quickly mobilise specialist community provision, perhaps with the local in-patient secure unit acting as a ‘hub’ and a source of specialist trained staff.

Nearly all of the specialist community teams included psychiatry, psychology and nursing as core members of the team, with most of the teams also incorporating occupational therapists and speech and language therapists. The range of professionals employed in such teams is likely to be related to the complexity and multiple needs that people with intellectual disability and/or autism and offending behaviour present, and the additional needs of these populations in terms of optimising daily living and communication skills. The workforce competency framework from Health Education England does not specify which professionals should be in these teams, but instead focuses on the competencies of teams to provide at a specific pathway point; for example, in accessing a service, delivering a therapeutic intervention, assessment, formulation and treatment planning.^8^

Different models of providing specialist forensic community forensic care for people with intellectual disability and/or autism have been described, but there remains a lack of formal evaluation and no one model of service provision has emerged as clearly superior.^12–15^ Teams may be either Trust/Board-based or span a larger area, likely aligned with Provider Collaboratives in England, and there was a roughly equal split between these models in our survey. Specialist community forensic teams work differently and may be engaged in (a) providing advice and consultation to other teams/professionals; (b) engaging in direct work with people with an intellectual disability and/or autism, but not assuming statutory responsibility for patient care; and (c) having statutory responsibility for the person's mental healthcare, including responsibilities under the relevant mental health legislation. All of the specialist teams that responded to our survey fulfilled functions (a) and (b), and around two-thirds also fulfilled model (c), at least for some of their patients. Those teams that were Trust-based (as opposed to regional) were more likely to provide a care coordination function; there may be logistical and governance issues with a team that spans a wide area and different organisations assuming statutory responsibility for patients.

Perhaps unsurprisingly, a range of risk assessment and management approaches were used within Trusts/Boards for people with intellectual disability and/or autism and forensic needs. This may reflect the plethora of available tools and a lack of evidence, especially in people with neurodevelopmental disorders. The question of who is responsible for patients with intellectual disability and/or autism and forensic needs in a crisis is pertinent because of the complex and sometimes risky nature of this patient group, and the coordinated multi-agency response that is often required; for example, including mental health services, governmental justice departments, and the police or probation services. That specialist services that provided care coordination were more likely to assume responsibility in a crisis is perhaps unsurprising, as detailed knowledge of a patient and their relapse signature and risk factors is vital for providing safe and effective crisis care.

Our findings suggest that generic services (either intellectual disability community teams or mainstream forensic teams) do not appear to be well equipped to manage risks or clinical correlates in this population, with referral criteria often out of reach, or a lack of expertise or ability to adapt therapeutic programmes and approaches. Trusts/Boards that had access to specialist forensic intellectual disability and/or autism community teams were more likely to co-produce care plans with patients (an important element of patient-centred care) and were more likely to offer offence-specific psychotherapeutic interventions than those without specialist teams, which indicates the added value of such provision, although the impact of this on more concrete outcomes (e.g. recidivism) was not measured in the current study.

Where no specialist community forensic service exists, the responsibility to manage people with intellectual disability and/or autism rests with generic community forensic or intellectual disability services. There is an evident tension between mainstream and specialist services, and this can be a potent barrier against ensuring people receive the right support or are discharged from in-patient services in a timely manner. This study highlights a disparity in provision for people with intellectual disability or autism with forensic needs in the community, both across the nations of the UK and between areas within the same policy jurisdiction. Long-term follow-up or care coordination for patients in the community is a rarity, and formal arrangements between specialist community teams and their interfacing partners is often haphazard and limited.

### Lack of in-patient beds and suitable community placements

Although in-patient beds and community placements appear to be ostensibly unconnected, distinct entities, survey respondents identified that there is an important synergy between them. The provision of good quality, well-managed, suitable community placements are in short supply, leading to delays in discharge from hospital, and responsive care teams with good, experienced managers, working in concert with specialist teams are required to expedite discharges and avoid unnecessary admissions. There was a sense from some respondents that commissioners sought the avoidance of in-patient admission for people in crisis at all costs. There was a fear that delays in admission could introduce significant risks in terms of placement breakdown and risk to the public, whereas a strategic admission with specialist community team support could effectively manage a crisis and save the longer-term viability of precious community placements.

### Interface with the criminal justice system and legal frameworks

Liaison and Diversion teams in police custody and courts aim to divert vulnerable offenders to suitable community services rather than remand in custody. Notwithstanding the challenges of finding and sustaining such robust community support, there remains need to improve such services for those with neurodevelopmental conditions.^16,17^ A lack of capacity within criminal justice services for this group of offenders was reported especially around identification in the court setting and for those going into prison with a lack of community support on release. Defendants with intellectual disability presenting to court have significant risks of suicide and self-harm and higher rates of psychiatric comorbidity.^18^ Going forward, consideration needs to be afforded to how specialist forensic community teams work with other agencies such as housing, probation, prison and courts to ensure people with intellectual disability and autism are identified, but also supported in the community to reduce their risk of reoffending and address any ongoing health needs.

The issue of whether the current legal frameworks are sufficient to support people in the community was also highlighted. There has been a recent review of the Mental Health Act for England and Wales,^19^ and there is an ongoing review of mental health legislation in Scotland,^20^ both of which have discussed taking steps to reduce detentions on the grounds of intellectual disability and autism. However, concerns have been expressed about the unintended consequences of such reforms leading to more people with intellectual disability or autism going to prison or leaving high-risk individuals in the community without adequate support.^21^ The current legal framework remains a challenging arena in respect of the ongoing supervision of high-risk individuals in the community regardless of the provision of community-based specialist forensic teams. This issue is amplified in England and Wales, where patients subject to restriction with capacity to consent to care arrangements are currently unable to be conditionally discharged where their supervision would comprise an objective deprivation of liberty.^22^

There are myriad issues across care and criminal justice system pathways, and a comprehensive overview of the current state of evidence can be found in a recently published book that brings these themes together.^23^ In summary, services need to be joined up with an equity of access across the country/nations to avoid a ‘postcode lottery’ of services. This is crucial to provide proper care and optimal outcomes for our patients and their families and, ultimately, for society as a whole.

### Strengths and limitations

This survey provides the first national-level evidence of the provision of community services for people with intellectual disability and/or autism and forensic needs in the UK. The findings go some way to mapping the current service offered, and are an initial step in building an evidence base to inform further service development. The overall survey response rate of over 60% (over 85% for Scottish Health Boards) compares favourably with other studies using similar methodology; doctors’ response rates to surveys have been found to average at best 53%, with lower response rates reported for online surveys.^24^ That our survey was short and relatively quick to complete may have contributed to the high response rate, but necessarily limited the number and range of questions we could include.

The study had some limitations. We did not collect information about the clinical background of those who completed the survey, but did request that this was a senior clinician with sufficient knowledge of the local services; however, we did not triangulate data and were not able to check the accuracy of responses. We did not receive responses from every Trust/Health Board; Trusts or Health Boards without specialist community forensic services may have been less likely to respond, thus skewing our findings. Face-to-face interviews may have allowed for more in-depth responses and for clarification on the wording of questions.

### Future directions for research

Despite tensions between mainstream and specialist services being evident and, at times, fragmentation in the commissioning of services resulting in individuals falling through gaps in services, this national survey indicates that progress is being made in delivering community forensic services for people with intellectual disability and autism in keeping with national policy commenced over a decade ago.

The next steps in evaluating specialist forensic community teams for people with intellectual disability and/or autism are to test outcomes between the different types of teams. These would include clinical outcomes in terms of rates of readmission, recidivism and patient satisfaction with community support. There also needs to be a better understanding of the health economic correlate of these services, and whether there is cost-effectiveness of such services.

Given the large variation in the provision and size of specialist forensic community teams for people with intellectual disability and/or autism, there needs to be a better understanding of the impact of the size of the geographical area served and the socioeconomic deprivation characteristics on service provision and patient outcome. This will require an in-depth study that should include patients and families’ views on service provision.^25^

There is also a need to understand and acknowledge that people with intellectual disability and autism spectrum conditions are overlapping but distinct populations. Research therefore needs to prioritise the investigation of differential approaches and outcomes; this should lead to more nuanced and individualised approaches to care all the way along community and in-patient pathways, rather than current one size fits all approaches.

## Supporting information

McKinnon et al. supplementary material McKinnon et al. supplementary material

## Data Availability

The data that support the findings of this study are available from the corresponding author, I.M., upon reasonable request.
