# Supplementary material for: A UK-wide survey of community forensic services for adults with intellectual disability and/or autism
Source: BJPsych Open. 2024 Aug 20;10(5):e148. doi: 10.1192/bjo.2024.734 (PMC11698153; doi:10.1192/bjo.2024.734)
Supplement: McKinnon et al. supplementary material [file S2056472424007348sup001.docx]

# Supplementary material - RCPsych Forensic ID Subgroup - Community Forensic Services for people with Intellectual Disability or Autism. Online questionnaire.

Thank you for taking the time to complete our survey.

We have kept it short, and it covers just two pages. The responses will help the organisers of health services to improve access to specialist community support for patients with intellectual disability or autism with forensic needs. The survey will be open until 31 March 2023, 5pm.

***The information you provide will be used solely for the purposes of this project, and no identifiable information will be published. No patient level data are to be collected.***

If you have any queries please contact us at either: [iain.mckinnon@ncl.ac.uk](mailto:iain.mckinnon@ncl.ac.uk) or [rory.sheehan@kcl.ac.uk](mailto:rory.sheehan@kcl.ac.uk)

If you are happy to proceed then please continue with the survey by pressing "Next" below.

Thanks

Iain McKinnon, Jane McCarthy and Rory Sheehan on behalf of the RCPsych Forensic ID Special Interest Group.

1. What is the name of your NHS Trust / Health Board? *Required

FREE TEXT BOX

2. Which specialist inpatient services do you have within your trust/board that cater specifically for patients with Intellectual Disability (ID) or Autism? Please select all that apply. *Required

| High secure | Medium secure | Low secure |
| --- | --- | --- |
| Locked rehabilitation | Assessment and treatment unit | No specific ID or autism beds |

2a. If you do not have specific beds for people with ID or Autism, what arrangements do you have should such a patient require admission?

FREE TEXT BOX

3. What teams does your Trust/Board have for the management of patients with ID or autism with forensic needs in the community? Please select all that apply. *Required

| Forensic ID or autism community team | Generic ID community team | Generic autism community team |
| --- | --- | --- |
| Generic forensic community team | Generic community mental health team | None  Skip 3a-c and go to 3d |

3a. Which professionals do you have in your forensic community ID or autism team. Please select all that apply.

| Community nurses | Psychiatry | Psychology |
| --- | --- | --- |
| Social worker | Specialty support worker | Peer support worker |
| Dietician | Speech and language therapist | Occupational therapist |
| Other | | |

3ai. If you selected Other, please specify:

FREE TEXT BOX

3b. Within your forensic community ID or autism team which of the following do you provide? Please select all that apply.

| Advice and consultation to another team with primary responsibility | Direct work with patients (including direct assessment and treatment) | Care coordination |
| --- | --- | --- |
| Provision of a Responsible Clinician/RMO | Other | |

3b1. If you selected Other, please specify:

FREE TEXT BOX

3c. Is your forensic community ID or autism team based within your Trust/Board, or spread across a wider multiple Trust/Board area?

| Single Trust/Board area | Across multiple Trust/Board areas |
| --- | --- |

3ci. If your forensic community ID or autism team is spread across a wider multiple Trust/Board area, which Trusts/Boards do you work in partnership with?

FREE TEXT BOX

3d. If you do not have any of these teams, what arrangements do you have to support patients with ID or autism with forensic needs in the community?

FREE TEXT BOX

4. Who is responsible for the care coordination for patients with ID or autism with forensic needs in the community? Please select all that apply. * Required

| Forensic ID or autism community team | Generic ID community team | Generic autism community team |
| --- | --- | --- |
| Generic forensic community team | Generic community mental health team | Other |

4a. If you selected Other, please specify:

FREE TEXT BOX

4b. Feel free to expand on these arrangements:

FREE TEXT BOX

5. What processes do you use for assessing and managing risk in patients with ID or autism with forensic needs in the community? Please select all that apply. * Required

| MDT reviews | Review of structured risk assessments | Care programme approach |
| --- | --- | --- |
| Case conference review meetings | Care and treatment reviews | Multi-agency meetings (e.g. MAPPA, MARAC) |
| Other | | |

5a. If you selected Other, please specify

FREE TEXT BOX

6. Are your patients' care plans co-produced? * Required

| Yes | No |
| --- | --- |

6a. If pathways are co-produced, what tools do you employ (e.g. recovery star, my shared pathway, etc.)?

FREE TEXT BOX

6b. If No, do you have any plans in place to co-produce care pathways, and what are they?

FREE TEXT BOX

7. How does your Trust/Board support patients with ID or autism with forensic needs in a crisis, emergency or recall situation? Please select all that apply. * Required

| Assessment and management provided by specialist forensic team | Scaffolding by specialist forensic team but management carried out by general teams | Support provided by general team with no input from specialist forensic team |
| --- | --- | --- |
| Other | | |

7a. If you selected Other, please specify:

FREE TEXT BOX

8. Can patients with ID or autism with forensic needs access psychotherapeutic interventions in the community? Please select all that apply. * Required

| Yes - offence related | Yes - for mental health | No |
| --- | --- | --- |

8a. Which team would offer offence related psychotherapeutic interventions? Please select all that apply.

| Specialist forensic team | Generic community ID team | Forensic community MH team |
| --- | --- | --- |
| Inpatient teams | Other | |

8ai. If you selected Other, please specify:

FREE TEXT BOX

8b. Which team provides mental health related psychotherapeutic interventions? Please select all that apply.

| Specialist forensic team | Generic community ID team | Forensic community MH team |
| --- | --- | --- |
| Inpatient teams | Other | |

8bi. If you selected Other, please specify:

FREE TEXT BOX

9. Finally, we would like to know anything you would like to say about community provision for people with ID or autism with forensic needs. In particular, we would like to know about the specific challenges you have in providing community care for these particular patients. Thankyou!

FREE TEXT BOX

***Thank you for taking the time to complete our survey.***

***If you have any queries please contact us at either:*** [***iain.mckinnon@ncl.ac.uk***](mailto:iain.mckinnon@ncl.ac.uk) ***or*** [***rory.sheehan@kcl.ac.uk***](mailto:rory.sheehan@kcl.ac.uk)
